# Self-reported hearing-related symptoms among adults with diabetes: a cross-sectional study

**DOI:** 10.3389/fpubh.2026.1943385

**Published:** 2026-10-28

**Authors:** Beata Stepanow, Joanna Partyka, Weronika Ciszak, Anna Marcinkowska-Gapińska, Piotr Wolski, Katarzyna Karpus, Dagna Rudnik, Elzbieta Krupińska, Leszek Czupryniak

**Affiliations:** 1 Centre for Education and Specialized Medical Care, Kleczew, Poland; 2 Polish Diabetes Education Association, Kleczew, Poland; 3 Metabolica Sp. z o.o., Tarnów, Poland; 4 Department of Biophysics, Poznan University of Medical Sciences, Poznań, Poland; 5 Gemini ProMed Medical Center Sp. z o.o., Chojnice, Poland; 6 Department of Nursing, Jan Długosz University in Częstochowa, Częstochowa, Poland; 7 Department of Diabetology and Internal Medicine, Medical University of Warsaw, Warsaw, Poland

**Keywords:** adults with diabetes, diabetes mellitus, hearing assessment, hearing impairment, hearing loss, hearing-related symptom score, listening-related fatigue, self-reported hearing-related symptoms

## Abstract

**Introduction:**

Hearing impairment has been increasingly recognized as being associated with diabetes mellitus. Although previous studies have demonstrated a higher prevalence of hearing loss among people with diabetes, less is known about self-reported hearing-related symptoms and their association with demographic and clinical characteristics among adults with diabetes.

**Purpose:**

To assess the burden of self-reported hearing-related symptoms among adults with diabetes and examine their associations with age, sex, and diabetes duration.

**Methods:**

A cross-sectional study was conducted during the first half of 2022 among 128 adults with diabetes. Data were collected using an author-developed questionnaire constructed based on a review of the literature addressing hearing-related symptoms and their everyday and social consequences. The questionnaire was not formally validated. A Hearing-Related Symptom Score was calculated from seven self-reported symptom questions (range, 0–7). Multivariable Poisson regression with robust standard errors was used to examine associations between the Hearing-Related Symptom Score and age, sex, and diabetes duration.

**Results:**

The mean age was 51.19 years (SD, 16.88), and the median Hearing-Related Symptom Score was 4 (range, 0–7). In the multivariable model (*n* = 127), older age was independently associated with a higher symptom score (adjusted ratio of expected symptom-score burden, 1.02; 95% CI, 1.01–1.03; *P* < 0.001), while male sex was associated with a lower symptom score than female sex (adjusted ratio of expected symptom-score burden, 0.83; 95% CI, 0.69–0.99; *P* = 0.037). Diabetes duration was not independently associated with symptom score (adjusted ratio of expected symptom-score burden, 1.01; 95% CI, 1.00–1.02; *P* = 0.170). Age remained positively associated with symptom burden in both women and men; however, the age-by-sex interaction was not statistically significant (*P* = 0.501).

**Conclusion:**

Self-reported hearing-related symptom burden was associated with age and sex, but not independently with diabetes duration after adjustment. Findings should be interpreted cautiously given the cross-sectional design, convenience sampling, self-reported data, and use of a non-validated questionnaire. Further studies using validated instruments and objective hearing assessment are warranted.

## Introduction

Diabetes mellitus is a major public health challenge and requires ongoing patient engagement in everyday disease management. Effective diabetes care depends on patients' ability to participate in consultations, receive and understand health information, and make decisions about their care. Sensory impairments that interfere with everyday communication may therefore represent an additional challenge for people living with diabetes (1). Hearing impairment has received increasing attention in diabetes research, with growing evidence of an association between diabetes and hearing loss. Recent systematic reviews and meta-analyses have reported a higher prevalence of objectively assessed hearing loss among people with diabetes than among individuals without diabetes. Age and diabetes duration have also been identified as potential factors associated with hearing loss in people with diabetes (2, 3). However, most available studies have focused on clinically diagnosed or audiometrically measured hearing loss rather than on hearing-related symptoms experienced in everyday life. Hearing-related difficulties are not limited to reduced auditory sensitivity and may be experienced as problems in everyday communication and social participation. Individuals with hearing difficulties may have difficulty understanding speech in noisy environments, need to ask others to repeat information, and increase the volume of television or other audio devices (4, 5). Hearing difficulties may also require greater listening effort and may be accompanied by listening-related fatigue, although the available evidence remains heterogeneous (5, 6). Tinnitus has also been associated with diabetes in observational studies and meta-analytic evidence (7). Difficulties with hearing and communication may further affect participation in social activities and contribute to social withdrawal or isolation (8).

Despite the growing evidence on clinically assessed hearing loss in people with diabetes, less is known about the burden of self-reported hearing-related symptoms in everyday life, particularly among individuals without a prior self-reported diagnosis of hearing loss. Characterizing these experiences may complement evidence based on clinically assessed hearing loss and may help define priorities for future research. However, symptoms such as listening-related fatigue and avoidance of social situations may also reflect factors other than hearing difficulties, including depression, anxiety, cognitive function, or reduced mobility. Therefore, their interpretation requires caution. In this context, the present study aimed to assess the burden of self-reported hearing-related symptoms among adults with diabetes and examine their associations with age, sex, and diabetes duration.

## Materials and methods

### Study design and participants

A cross-sectional study was conducted during the first half of 2022 among adults with diabetes in Poland. The study was reported in accordance with the Strengthening the Reporting of Observational Studies in Epidemiology (STROBE) Statement for cross-sectional studies. The study was developed by the Polish Diabetes Education Association in collaboration with the Coalition “To Help the Dependent.”

Participants were recruited during diabetes education activities organized by the Polish Diabetes Education Association. Data collection was conducted during a single 1-week period across eight of the 16 voivodeships in Poland. A convenience sampling strategy was used.

Eligible participants were aged 18 years or older and had a self-reported diagnosis of diabetes mellitus, regardless of diabetes type. A total of 128 eligible participants were approached and invited to participate; all 128 agreed to participate and completed the questionnaire. No questionnaires were incomplete. One participant was unable to report the duration of diabetes because they did not remember it; therefore, analyses including diabetes duration were based on 127 participants.

No formal sample size calculation was performed because the study was exploratory and used a convenience sample.

### Data collection

The development of the questionnaire and the standardized data-collection procedure are presented in Figure 1. The questionnaire was developed by the study co-authors based on a review of published literature describing hearing-related experiences and their everyday communicative, functional, and social consequences, together with clinical and educational experience in diabetes care. The selected domains included everyday difficulties in hearing and understanding speech, difficulty having conversations in crowded places, the need to ask others to repeat information, increasing the volume of television or other audio devices, tinnitus, listening-related fatigue, and avoidance of social situations related to hearing difficulties. These domains were operationalized by the authors as seven yes/no questions for exploratory assessment of patient-reported hearing-related symptom burden. A brief study-specific questionnaire was used to allow assessment of these experiences within the limited time available during routine diabetes education visits, while covering the main hearing-related difficulties identified in the literature. The questionnaire was pre-tested for clarity and comprehensibility in two adults with diabetes and subsequently administered by 17 trained diabetes nurse educators during the 1-week data-collection period.

**Figure 1:**
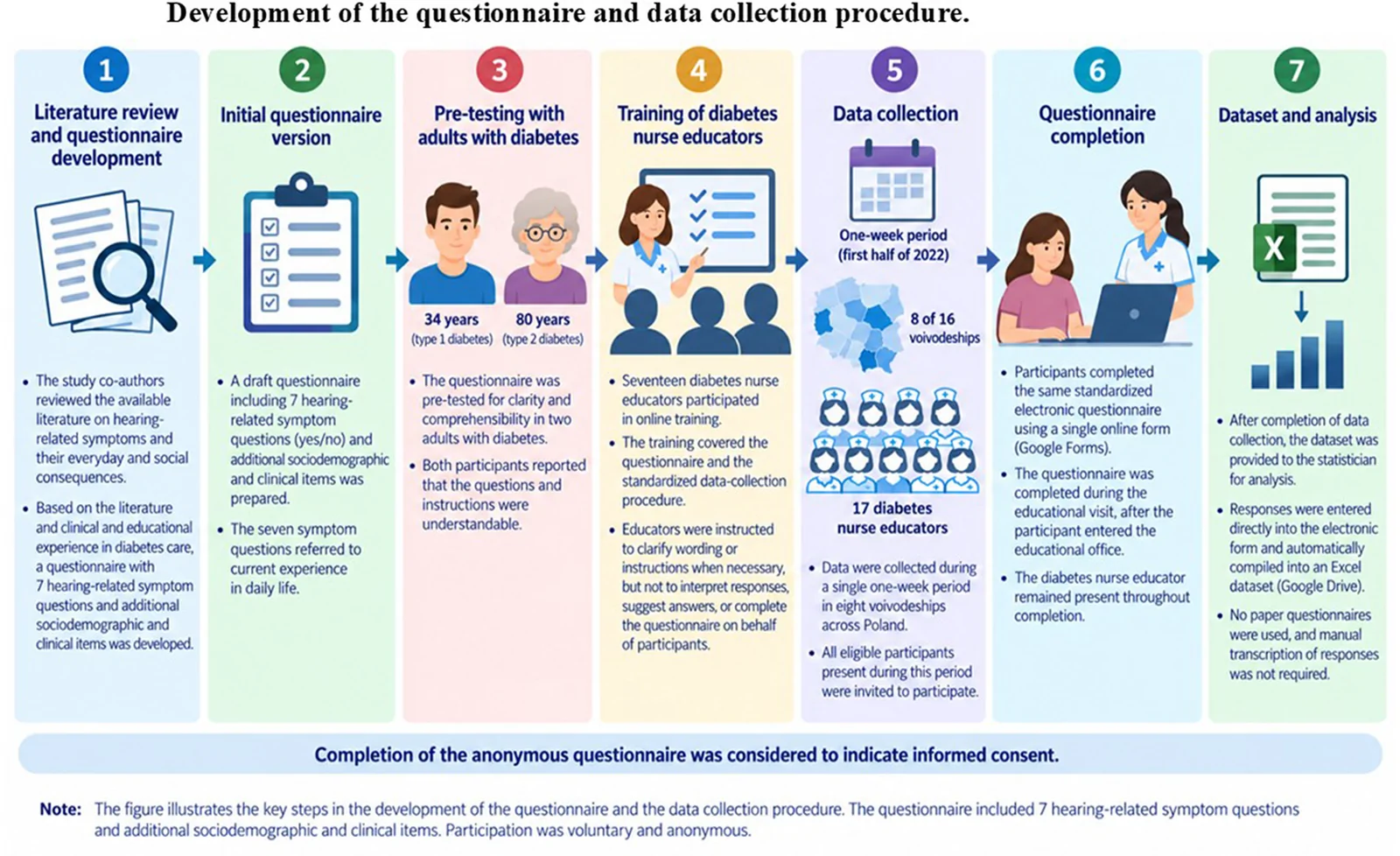
Development of the questionnaire and data collection procedure. The figure illustrates the key steps in the development of the questionnaire and the data collection procedure. The questionnaire included 7 hearing-related symptom questions and additional sociodemographic and clinical items. Participation was voluntary and anonymous.

Participants were recruited during routine diabetes education activities conducted by 17 diabetes nurse educators across eight of the 16 voivodeships in Poland. Data collection was conducted simultaneously during a single 1-week period (five working days). Recruitment took place during routine educational visits rather than during predefined study-specific recruitment sessions. All eligible adults with diabetes who attended an educational visit during the data-collection period were invited to participate.

Other individuals attending the educational activities who did not have diabetes were not eligible for the study and were therefore not invited to participate.

All participants used the same standardized electronic questionnaire administered through a single online form (Google Forms). The questionnaire was completed during the educational visit in the presence of a diabetes nurse educator. Educators could clarify the wording or instructions when necessary but were instructed not to interpret responses, suggest answers, or complete the questionnaire on behalf of the participant. Educator presence was standardized for all participants; therefore, no separate assisted and unassisted groups were available for comparison or sensitivity analysis. Responses were entered directly into the electronic form and automatically compiled into an Excel dataset. No paper questionnaires were used and no manual transcription of responses was required. After completion of data collection, the dataset was provided to the statistician for analysis.

The questionnaire included items concerning age, sex, type of diabetes, diabetes duration, comorbid conditions, previous hearing examination, diagnosed hearing loss, use of hearing-assistive devices, and hearing-related symptoms. The structure of the questionnaire and the construction of the Hearing-Related Symptom Score are presented in Figure 2. The full questionnaire used for data collection is provided as Supplementary material 1.

**Figure 2:**
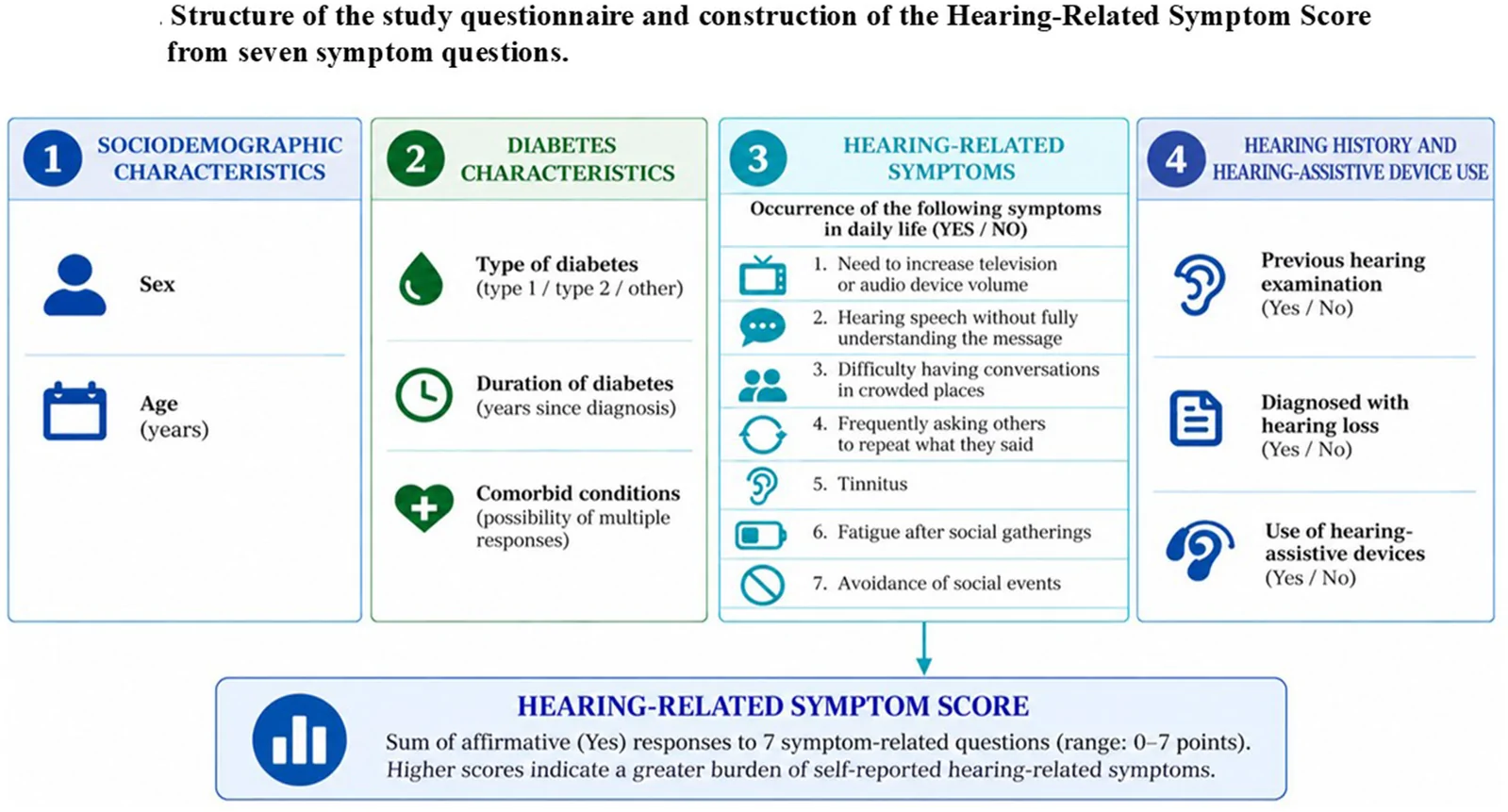
Structure of the study questionnaire and construction of the Hearing-Related Symptom Score from seven symptom questions.

### Assessment of hearing-related symptoms

Before the study, the questionnaire was pre-tested for clarity and comprehensibility in two adults with diabetes: one 34-year-old person with type 1 diabetes and one 80-year-old person with type 2 diabetes. Both participants reported that the questions and instructions were understandable. This pre-testing was intended to identify potential problems with wording and comprehension and did not constitute formal cognitive testing or psychometric validation.

The seven hearing-related symptom questions referred to the participant's current experience and were presented as mandatory yes/no questions in the electronic form. All participants answered all seven questions. The questions addressed the need to increase the volume of television or other audio devices, difficulty understanding spoken messages, difficulty having conversations in crowded places, frequently asking others to repeat information, tinnitus, fatigue after social gatherings, and avoidance of social events.

An affirmative response (“yes”) was assigned 1 point and a negative response (“no”) 0 points. The Hearing-Related Symptom Score therefore ranged from 0 to 7, with higher scores indicating a greater burden of self-reported hearing-related symptoms.

The questionnaire was developed specifically for this study and has not undergone formal psychometric validation. No formal cognitive testing was performed beyond the pre-testing for clarity described above, and no internal consistency analysis, including Cronbach's alpha, was performed. Accordingly, the Hearing-Related Symptom Score was used as an exploratory measure of self-reported symptom burden and should not be interpreted as a validated diagnostic or screening instrument. The questions were intended to capture patient-reported experiences that could facilitate discussion of hearing-related difficulties during diabetes care, rather than to diagnose hearing loss.

Following the symptom questions, participants were asked about previous hearing examinations and whether they had a previously diagnosed hearing loss. The questionnaire instructed participants who reported no previous hearing examination and at least one hearing-related symptom to seek further consultation with a hearing-aid specialist.

### Additional variables

The study collected information on age, sex, type of diabetes, duration of diabetes, depression, previous hearing examinations, self-reported previous diagnosis of hearing loss, use of hearing-assistive devices, and selected comorbid conditions. These data were obtained from participants through the electronic questionnaire.

Depression was based on self-reported information provided by participants in the questionnaire and was not established using a standardized diagnostic interview or independently verified through medical records.

For the purposes of describing multimorbidity, the number of reported comorbid conditions was calculated from hypertension, cardiovascular disease, thyroid disease, and depression. Diabetes and hearing loss were not counted as comorbid conditions because they were the conditions under study.

All information concerning participants' medical history and previous hearing-related assessments was self-reported. No medical records or objective audiometric measurements were used to verify these reports.

### Statistical analysis

Continuous variables were summarized using the mean and standard deviation (SD) and, where appropriate, the median and range. Categorical variables were summarized using numbers and percentages. The primary outcome was the Hearing-Related Symptom Score, ranging from 0 to 7 and calculated as the number of affirmative responses to seven binary symptom questions. Because the outcome represents a non-negative integer-valued, count-type measure of self-reported symptom burden, a multivariable Poisson regression model with robust standard errors was used as a parsimonious log-link approach. The bounded nature of the score (0–7) was taken into account in the interpretation, and the score was not assumed to represent a conventional unbounded Poisson count. The exponentiated regression coefficients were interpreted as multiplicative ratios of the expected Hearing-Related Symptom Score.

The model included age, sex, and diabetes duration as pre-specified covariates. Depression and selected comorbid conditions were collected as additional clinical characteristics but were not pre-specified as covariates in the primary regression model. Age and diabetes duration were entered as continuous variables, with effect estimates corresponding to a 1-year increase. Sex was entered as a binary variable, with women as the reference category. Because age was considered a potential confounding factor and a possible source of differences between women and men, additional analyses were performed to examine the association between age and symptom burden within each sex. Sex-stratified Poisson regression models with robust standard errors included age and diabetes duration as covariates. An age-by-sex interaction term was additionally tested in the multivariable model to assess whether the association between age and symptom burden differed between women and men.

The association between diabetes duration and the Hearing-Related Symptom Score was additionally explored using Spearman's rank correlation coefficient. Categorical variables were compared using the Pearson χ^2^ test, and differences in symptom scores between groups were assessed using the Mann–Whitney U test where appropriate.

A two-sided *P* value of < 0.05 was considered statistically significant. No formal correction for multiple comparisons was applied. The analyses were reassessed in collaboration with a statistician, with particular attention to potential confounding by age and to the robustness of the associations observed in the original analyses.

Statistical analyses were performed using STATISTICA software (TIBCO Software Inc., Palo Alto, CA, USA).

### Ethical considerations

Participation in the study was voluntary and anonymous. Participants were informed about the purpose of the study and their right to decline participation or withdraw from the survey at any time. Completion of the anonymous questionnaire was considered to indicate informed consent. No personally identifiable information was collected, and all analyses were conducted using anonymized data. The study was designed and conducted as an anonymous, non-interventional questionnaire study and did not constitute a medical experiment. It involved no medical intervention, modification of clinical care, collection of biological material, or access to medical records. Under the applicable Polish regulatory framework, formal approval by a research ethics committee was not required for this type of study.

### Patient and public involvement

The survey was developed and implemented as part of an educational and public health initiative conducted by the Polish Diabetes Education Association in collaboration with the Coalition “To Help the Dependent.” Diabetes educators were involved in the development of the questionnaire and supported participant engagement during data collection.

### Data availability statement

The datasets generated and analyzed during the current study are not publicly available because they contain information that could compromise participant confidentiality. The datasets are available from the corresponding author upon reasonable request.

## Results

### Participant characteristics

A total of 128 adults with diabetes participated in the study. The mean age was 51.19 years (SD, 16.88). Women comprised 62.5% of the study population (80/128), and men 37.5% (48/128). Type 1 diabetes was reported by 58 participants (45.3%), type 2 diabetes by 66 (51.6%), and other types of diabetes by 4 (3.1%). The most frequently reported comorbid condition was hypertension (64.1%), followed by thyroid disease (41.4%), cardiovascular disease (39.8%), and depression (25.8%). A self-reported prior diagnosis of hearing loss was reported by 35 participants (27.3%). Detailed sociodemographic and clinical characteristics are presented in Table 1.

**Table 1:** Sociodemographic and clinical characteristics of the study population (*N* = 128).

Variable	*n*	%
Sex		
Female	80	62.5
Male	48	37.5
Age (years)		
18–26	8	6.3
27–35	19	14.8
36–45	19	14.8
46–55	28	21.9
56–65	26	20.3
66–75	14	10.9
76–85	13	10.2
>85	1	0.8
Type of diabetes		
Type 1 diabetes	58	45.3
Type 2 diabetes	66	51.6
Other type of diabetes	4	3.1
Selected comorbidities		
Hypertension	82	64.1
Thyroid disease	53	41.4
Cardiovascular disease	51	39.8
Depression	33	25.8
Self-reported prior diagnosis of hearing loss	35	27.3

### Hearing-related symptom burden

The Hearing-Related Symptom Score had a mean of 3.35 (SD, 2.17), a median of 4, and a range of 0–7. The most frequently observed score was 4, reported by 37 participants (28.9%). A score of 0 was reported by 23 participants (18.0%), whereas 14 participants (10.9%) had the maximum score of 7. The complete distribution of the Hearing-Related Symptom Score is presented in Table 2.

**Table 2:** Distribution of the hearing-related symptom score in the study population (*N* = 128).

Hearing-related symptom score	*n*	%
0	23	18.0
1	8	6.3
2	9	7.0
3	16	12.5
4	37	28.9
5	17	13.3
6	4	3.1
7	14	10.9
Total	128	100.0

*n*, number of participants; %, percentage of the study population. The Hearing-Related Symptom Score was calculated as the sum of seven self-reported hearing-related symptoms, with 1 point assigned for each “yes” response and 0 points for each “no” response; total scores ranged from 0 to 7, with higher scores indicating a greater burden of hearing-related symptoms. The mean score was 3.35 ± 2.17, the median was 4, and the range was 0–7.

The most frequently reported individual symptoms were difficulty having conversations in crowded places (69.5%), frequently asking others to repeat what they had said (68.8%), and difficulty understanding spoken messages (64.8%). Tinnitus was reported by 58.6% of participants, and 57.0% reported a need to increase the volume of television or other audio devices. Fatigue after social gatherings was reported by 47.7% of participants, while 23.4% reported avoiding social events.

### Hearing history and hearing-assistive device use

Among the 128 participants, 73 (57.0%) reported having previously undergone a hearing examination. A self-reported prior diagnosis of hearing loss was reported by 35 participants (27.3%). Among these 35 participants, 11 (31.4%) reported using hearing-assistive devices, while 24 (68.6%) reported not using them. These findings are presented in Table 3.

**Table 3:** Self-reported hearing history and hearing-related care in the study population (*N* = 128).

Parameter	*n*	%
Previous hearing examination	73	57.0
Self-reported prior diagnosis of hearing loss	35	27.3
Use of hearing-assistive devices among participants with a prior self-reported diagnosis of hearing loss (n = 35)	11	31.4

*n*, number of participants; %, percentage. All information was self-reported. Among the 35 participants who reported a prior diagnosis of hearing loss, 11 reported using hearing-assistive devices and 24 reported not using them.

Factors Associated With Hearing-Related Symptom Burden. In the multivariable Poisson regression model including age, sex, and diabetes duration (*n* = 127), age was independently associated with a higher Hearing-Related Symptom Score. For each 1-year increase in age, the adjusted ratio of the expected Hearing-Related Symptom Score was 1.02 (95% CI, 1.01–1.03; *P* < 0.001), corresponding to a 2.0% higher expected symptom-score burden. Male sex was associated with a lower symptom score compared with female sex (adjusted ratio of the expected Hearing-Related Symptom Score, 0.83; 95% CI, 0.69–0.99; *P* = 0.037). Diabetes duration was not independently associated with the Hearing-Related Symptom Score (adjusted ratio of the expected Hearing-Related Symptom Score, 1.01; 95% CI, 1.00–1.02; *P* = 0.170).

In the exploratory unadjusted analysis, diabetes duration showed a weak positive correlation with the Hearing-Related Symptom Score (Spearman r = 0.19; *P* = 0.03). However, after adjustment for age and sex in the multivariable model, diabetes duration was no longer independently associated with symptom burden.

In sex-stratified analyses, age remained positively associated with the Hearing-Related Symptom Score in both women and men. Among women (*n* = 79), the adjusted ratio of the expected Hearing-Related Symptom Score for each 1-year increase in age was 1.02 (95% CI, 1.01–1.03; *P* < 0.001). Among men (*n* = 48), the corresponding adjusted ratio was 1.03 (95% CI, 1.01–1.04; *P* < 0.001).

The age-by-sex interaction was not statistically significant (adjusted ratio of the expected Hearing-Related Symptom Score, 1.01; 95% CI, 0.99–1.02; *P* = 0.501). Given the relatively small number of men (*n* = 48), this finding should be interpreted cautiously and does not establish the absence of an age-by-sex interaction. Detailed results of the multivariable and sex-stratified analyses are presented in Table 4.

**Table 4:** Multivariable analysis of factors associated with the hearing-related symptom score.

Variable	Adjusted ratio of expected symptom-score burden	95% CI	*P* value
Main multivariable model (*N* = 127)			
Age, per 1-year increase	1.02	1.01–1.03	< 0.001
Male sex vs female sex	0.83	0.69–0.99	0.037
Diabetes duration, per 1-year increase	1.01	1.00–1.02	0.170
Additional analyses			
Age, per 1-year increase — women (n = 79)	1.02	1.01–1.03	< 0.001
Age, per 1-year increase — men (n = 48)	1.03	1.01–1.04	< 0.001
Age × sex interaction	1.01	0.99–1.02	0.501

CI, confidence interval. Adjusted estimates were obtained from multivariable Poisson regression models with robust standard errors. The exponentiated regression coefficients represent multiplicative ratios of the expected Hearing-Related Symptom Score (symptom-score burden). The main model included age, sex, and diabetes duration as covariates and included 127 participants with complete data for all model variables. The sex-stratified models included age and diabetes duration as covariates. The age × sex interaction was tested in a model including age, sex, and the age × sex interaction term. The Hearing-Related Symptom Score ranged from 0 to 7.

## Discussion

The present study provides a patient-reported perspective on hearing-related difficulties among adults with diabetes that complements the substantial literature on clinically diagnosed or objectively measured hearing impairment. The most important finding was the independent association between age and the burden of self-reported hearing-related symptoms. For each additional year of age, the expected burden of self-reported hearing-related symptoms was approximately 2% higher after adjustment for sex and diabetes duration, and this association remained significant in both women and men.

The age-by-sex interaction was not statistically significant; however, the study was not specifically powered to detect interaction effects. Therefore, the absence of a statistically significant interaction should not be interpreted as evidence that the association between age and symptom burden is definitively the same in women and men. The sex-stratified estimates, particularly among men (*n* = 48), should be interpreted cautiously because the relatively small subgroup size limits the precision and generalizability of these estimates. These findings are consistent with previous evidence identifying age as an important factor in hearing impairment among people with diabetes (2, 3, 9–11).

Importantly, however, our outcome was a measure of self-reported symptoms rather than clinically diagnosed or audiometrically confirmed hearing loss. The finding that diabetes duration was not independently associated with symptom burden after adjustment for age and sex is also noteworthy. Although a weak positive association was observed in the unadjusted analysis, it did not persist after adjustment, suggesting that the crude relationship between diabetes duration and symptom burden may partly reflect the contribution of age.

The individual symptom pattern provides further insight into the experiences reported by participants. Difficulty having conversations in crowded places, understanding spoken messages, and frequently asking others to repeat information were the most common symptoms. These experiences are consistent with the communication difficulties described in the hearing literature and with evidence that hearing impairment may increase listening effort, particularly during speech comprehension in challenging listening conditions (4, 5). Listening-related fatigue was reported by almost half of the participants, which is noteworthy in light of systematic-review evidence suggesting an association between hearing loss and fatigue, although the available evidence is heterogeneous (6). Tinnitus was also frequently reported and is consistent with recent meta-analytic evidence indicating an association between diabetes and tinnitus (7). Social avoidance was less frequent than the communication-related symptoms but remains relevant because hearing difficulties may be associated with reduced social participation, loneliness, and social isolation (8, 12, 13). At the same time, fatigue after social gatherings and avoidance of social situations are non-specific experiences. They may also be influenced by depression, anxiety, cognitive function, or reduced mobility. Previous research has demonstrated associations between hearing loss and depression and other psychosocial outcomes (14, 15), but these relationships do not establish that the corresponding symptoms observed in our study were caused by hearing difficulties.

An important contribution of this study is the focus on how hearing-related difficulties are experienced in everyday life rather than solely on whether a participant has previously received a hearing-loss diagnosis. More than half of the participants reported a previous hearing examination, whereas 27.3% reported a previous diagnosis of hearing loss, and only 31.4% of those with a previous diagnosis reported using hearing-assistive devices. These findings cannot be interpreted as evidence of undiagnosed hearing loss because hearing status was self-reported and no audiometric assessment was performed. Nevertheless, they illustrate that a person's experience of hearing difficulty may not be fully captured by the presence or absence of a previous diagnosis. This distinction is particularly relevant because hearing loss may develop gradually and may remain unrecognized or unmanaged (16). The seven questions used in the present study were based on domains described in previous literature concerning everyday communication difficulties, listening effort and fatigue, tinnitus, and social consequences of hearing difficulties (4–8, 12, 13), and were subsequently operationalized by the authors as an exploratory questionnaire. Thus, although the questionnaire was not formally validated, its content was grounded in previously described patient experiences rather than selected arbitrarily. The resulting Hearing-Related Symptom Score should nevertheless be regarded as exploratory and should not be interpreted as a validated scale, diagnostic measure, or screening instrument. The seven symptoms included in the score represent heterogeneous experiences and may not reflect a single underlying construct. The present findings therefore provide a basis for future work using validated patient-reported measures and objective hearing assessment.

These findings may have practical relevance for diabetes care. Recent literature has specifically highlighted the potential value of incorporating hearing health into diabetes care and considering hearing and communication needs within person-centered care (17). In this context, brief questions about understanding speech, coping with background noise, asking others to repeat information, increasing the volume of audio devices, tinnitus, listening-related fatigue, or withdrawal from social situations may provide a simple starting point for a conversation about hearing-related difficulties. Such questions should complement rather than replace objective hearing assessment. The present study did not evaluate diagnostic accuracy, sensitivity, specificity, predictive values, referral completion, treatment effects, or subsequent clinical outcomes. It therefore cannot establish that the questionnaire functions as a screening instrument or that asking these questions improves communication, diabetes education, self-management, adherence, or other health outcomes. The findings should also be interpreted in light of the cross-sectional design, self-reported medical and hearing history, absence of audiometric assessment, non-validated questionnaire, and convenience sampling. Further studies should combine validated patient-reported measures with objective hearing assessment to determine how everyday hearing-related symptoms correspond to clinically confirmed hearing impairment and whether systematic attention to these experiences has measurable clinical value.

## Conclusion

Self-reported hearing-related symptoms were common among adults with diabetes in this study.

Age was independently associated with greater symptom burden, and this association remained evident in both women and men. The age-by-sex interaction was not statistically significant, although the study was not specifically powered to detect interaction effects. In contrast, diabetes duration was not independently associated with symptom burden after adjustment for age and sex. The predominance of everyday communication difficulties, including problems understanding speech and having conversations in crowded places, highlights the value of considering patients' experiences of hearing difficulties alongside their previous hearing history. The seven-item questionnaire used in this study was an exploratory, non-validated measure and should not be considered a diagnostic or screening instrument. Further research using validated patient-reported measures together with objective hearing assessment is needed to clarify the relationship between self-reported hearing-related symptoms and clinically confirmed hearing impairment in people with diabetes.

## Data Availability

The datasets generated and analyzed during the current study are not publicly available because they contain information that could compromise participant confidentiality. The datasets are available from the corresponding author upon reasonable request.
