# Supplementary material for: Self-reported hearing-related symptoms among adults with diabetes: a cross-sectional study
Source: Front Public Health. 2026 Oct 28;14:1943385. doi: 10.3389/fpubh.2026.1943385 (PMC13752004; doi:10.3389/fpubh.2026.1943385)
Supplement: Supplementary file 1 [file Data_Sheet_1.pdf]

Supplementary Material 1. Study questionnaire used for data collection.

DIABETES AND HEARING-RELATED SYMPTOMS

1. SEX *

Select only one answer.

- FEMALE
- MALE

2. AGE IN YEARS *

Please enter your age in years.

________ years

3. WHAT TYPE OF DIABETES DO YOU HAVE? *

Select only one answer.

- Type 1
- Type 2
- Other: __________

4. HOW MANY YEARS HAVE YOU HAD DIABETES? *

(Alternatively, please indicate the year of diagnosis.)

________ years

5. PLEASE INDICATE THE COMORBID CONDITION(S) YOU HAVE. *

You may select more than one answer.

- Hypertension
- Depression
- Cardiovascular disease
- Thyroid disease
- Hearing loss
- I do not know
- I do not have any comorbid conditions

6. DO YOU NOTICE ANY OF THE FOLLOWING IN YOURSELF? *

Please answer each of the following questions.

	YES	NO
Do you need to set the television volume higher than other people (household members, neighbours, visitors)?	☐	☐
Does it often happen that you hear what other people say but do not necessarily understand what they are saying?	☐	☐
Do you often have difficulty having conversations in crowded places?	☐	☐
Do you often ask people you are talking to to repeat what they have said or to speak louder?	☐	☐
Do you experience ringing, whistling, buzzing or noise in your ears?	☐	☐
Do you feel tired after participating in social events?	☐	☐
Do you avoid social events?	☐	☐

7. HAVE YOU EVER HAD A HEARING EXAMINATION? *

Select only one answer.

- YES
- NO – if you answered YES to at least one item in Question 6, please consult a hearing-aid specialist.

8. HAVE YOU BEEN DIAGNOSED WITH HEARING LOSS? *

Select only one answer.

- YES – please proceed to Question 9.
- NO – thank you. This is the end of the questionnaire. Please click “SUBMIT”.

9. DO YOU USE HEARING-ASSISTIVE DEVICES?

Select only one answer.

- YES - thank you. This is the end of the questionnaire. Please click “SUBMIT”.
- NO - thank you. This is the end of the questionnaire. Please click “SUBMIT”.

---

* Required question – an answer is required to proceed to the next question.
